# Spatial variations in the incidence of breast cancer and potential risks associated with soil dioxin contamination in Midland, Saginaw, and Bay Counties, Michigan, USA

**DOI:** 10.1186/1476-069X-7-49

**Published:** 2008-10-21

**Authors:** Dajun Dai, Tonny J Oyana

**Affiliations:** 1 Environmental Resources and Policy Program, Southern Illinois University, 405 West Grand Avenue, MC 4637, Carbondale, IL 62901-4637, USA; 2 Department of Geography and Environmental Resources, Southern Illinois University, 1000 Faner Drive, MC 4514, Carbondale, IL 62901-4514, USA; 3 Department of Geography, Northern Illinois University, DeKalb, IL 60115, USA

## Abstract

**Background:**

High levels of dioxins in soil and higher-than-average body burdens of dioxins in local residents have been found in the city of Midland and the Tittabawassee River floodplain in Michigan. The objective of this study is threefold: (1) to evaluate dioxin levels in soils; (2) to evaluate the spatial variations in breast cancer incidence in Midland, Saginaw, and Bay Counties in Michigan; (3) to evaluate whether breast cancer rates are spatially associated with the dioxin contamination areas.

**Methods:**

We acquired 532 published soil dioxin data samples collected from 1995 to 2003 and data pertaining to female breast cancer cases (*n *= 4,604) at ZIP code level in Midland, Saginaw, and Bay Counties for years 1985 through 2002. Descriptive statistics and self-organizing map algorithm were used to evaluate dioxin levels in soils. Geographic information systems techniques, the Kulldorff's spatial and space-time scan statistics, and genetic algorithms were used to explore the variation in the incidence of breast cancer in space and space-time. Odds ratio and their corresponding 95% confidence intervals, with adjustment for age, were used to investigate a spatial association between breast cancer incidence and soil dioxin contamination.

**Results:**

High levels of dioxin in soils were observed in the city of Midland and the Tittabawassee River 100-year floodplain. After adjusting for age, we observed high breast cancer incidence rates and detected the presence of spatial clusters in the city of Midland, the confluence area of the Tittabawassee, and Saginaw Rivers. After accounting for spatiotemporal variations, we observed a spatial cluster of breast cancer incidence in Midland between 1985 and 1993. The odds ratio further suggests a statistically significant (*α *= 0.05) increased breast cancer rate as women get older, and a higher disease burden in Midland and the surrounding areas in close proximity to the dioxin contaminated areas.

**Conclusion:**

These findings suggest that increased breast cancer incidences are spatially associated with soil dioxin contamination. Aging is a substantial factor in the development of breast cancer. Findings can be used for heightened surveillance and education, as well as formulating new study hypotheses for further research.

## Background

**Figure 1:**
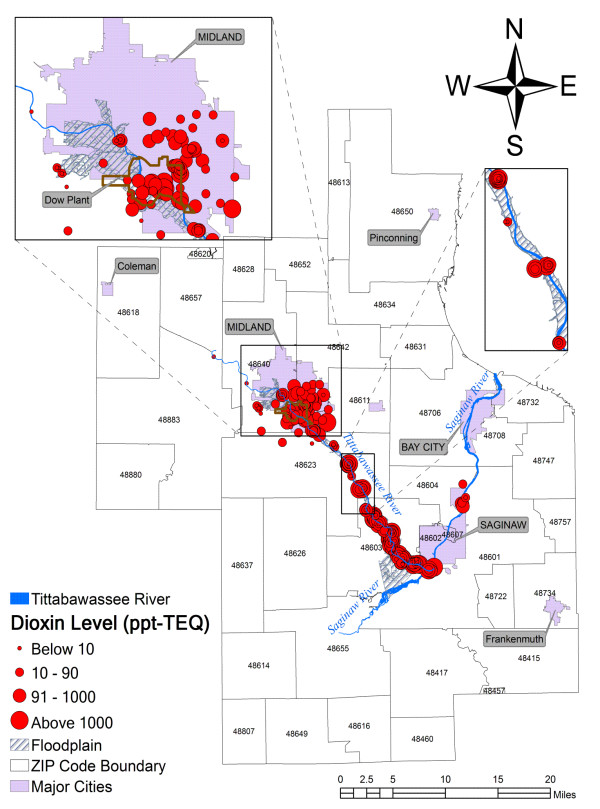
**Study area**. The study area shows sampling locations and corresponding dioxin levels, Tittabawassee River and its floodplain, and major cities. Michigan soil generic residential direct contact criterion for dioxins (RDCC) is 90 ppt TEQ.

Previous studies have reported higher than normal levels of dioxins in some locations in the city of Midland and Tittabawassee River floodplain in Michigan (Figure 1); while dioxin concentration in soils upstream of the river is similar to background levels across Michigan [1-5]. The most probable historic source of dioxins in the river is located in the city of Midland from industrial processes in the Dow Chemical Company's (Dow) Midland plant [2,3,6,7]. As by-products in chlorine-based chemical processes, dioxins were released into the air and water decades ago and accumulated in the sediments and soils in and near the Tittabawassee River [1,3]. Floods then swept and redeposited sediments and soils within the floodplain. Recent studies [8] found that living on property with soils contaminated by dioxins and eating fish from the Tittabawassee River, the Saginaw River, and Saginaw Bay led to higher levels of dioxins in people's blood. Inspired by the increased concern regarding the possible health effects, this study aimed at evaluating the soil dioxin contamination and exploring the potential risks associated with breast cancer incidence in the region.

Dioxin refers to 210 congeners/isomers of structurally and chemically related polychlorinated *dibenzo*-*para*-*dioxins *(PCDDs) and polychlorinated *dibenzofurans *(PCDFs), and the 2,3,7,8-tetra-CDD (TCDD) is considered the most toxic dioxin congener in this group [9,10]. A concept of toxic equivalency factors (TEFs) is used to compare the relative toxicity of other dioxin congeners with that of TCDD [10]. A total toxic equivalent (TEQ) is then determined by adding all dioxin congeners in a sample together on the basis of TEFs. Dioxins are persistent in the environment and resistant to biodegradation. The half-life of TCDD is 5.8 to 11.3 years in human body [11], 9 to 15 years in surface soil, and 25 to 100 years in subsurface soil[12]. People's exposure pathways to dioxins include inhalation, ingestion, and dermal contact [3,8]. TCDD has been classified as a human carcinogen [13] and has the potential to disrupt multiple endocrine pathways [14-16]. Studies have shown an apparent increase in the incidence of breast cancer [17-19] or the mortality rates of breast cancer [20,21] with dioxin exposure.

Breast cancer refers to cancerous tumors consisting of uncontrolled growth and spread of abnormal cells formed in breast tissues, usually ducts and lobules [22]. It is the most common cancer among women in the United States [23]. National breast cancer incidence experienced an apparent increase with annual percent change (APC) of 3.7 from 1980 to 1987, a slight increase from 1987 to 2001 (APC = 0.4), and a noticeable decline from 2001 to 2005 (APC = -3.1) [24]. In 2005, the annual incidence rate was 124.3 per 100,000 females [24]. Each year about $8.1 billion is spent on treatment of breast cancer in the United States [25]. Although the causal factors of breast cancer are not fully known, risks factors for developing the disease include history of cancer in one breast, family history of breast cancer, breast implants, history of benign breast disease, and exposure to endocrine disruption chemicals [22,26]. Among these risk factors, exposure to carcinogens, especially endocrine disruption chemicals, is a higher-than-average risk for females to develop breast cancer [14,15].

Previous studies show breast cancer risk increases with exposures to high levels of dioxins [15,17-21,27,28]. For example, two human epidemiological studies – the Hamburg cohort [18] and the Seveso women cohort [19] – found an apparent increase of breast cancer incidence with rising dioxin exposure after validating exposure levels using serum levels of dioxin. Other studies also reported increased breast cancer incidence [17] and mortality [20,21,28] with dioxin exposures. Dioxins act like hormone disruptors [14,17,29], which may explain the link between high body burden of dioxins and the increased incidence of breast cancer.

Breast cancer is a major burden in Midland, Saginaw, and Bay Counties, Michigan. Existing data from the Michigan Department of Community Health (MDCH) indicate that breast cancer was one of the highest cancer burdens in the three counties from 1985 through 2002 [30]. For example, thirteen percent of the total cancer cases in the three counties are breast cancer, after lung and bronchus cancer (14%) and prostate gland cancer (18%) [30]. Given the evidence from human epidemiological studies and animal studies, high incidence rates of breast cancer support the hypothesis that dioxin contamination in soils may contribute significantly to the etiology and exacerbation of the development of breast cancer in this region.

Despite a variety of studies [1-8] investigating the soil dioxin contamination in this area, the resulting health effects in the local communities are largely unknown. In particular, the spatial relation between soil dioxin contamination and risks of breast cancer development is still unclear. Other challenges persist, for example very few blood samples and only limited number of soil samples are available in part due to expensive testing for dioxins. Currently, one soil sample may cost up to $800 and one blood sample may cost between $1,200 and $1,500. The sparsity of samples and the inadequate sampling spread (Figure 1) hardly meet the requirement of conventional statistical, geostatistical, and epidemiological studies. Inspired by the challenge and the growing concern over the concurrent high breast cancer rates with high levels of dioxin in soils, we employed a variety of spatial and statistical techniques to evaluate dioxin levels in soils and analyzed whether there is a spatial association with the incidence of breast cancer. These techniques include Geographic Information System (GIS) mapping, descriptive statistics, self-organizing map algorithm (SOM) [31], odds ratio and their corresponding 95% confidence intervals, Kulldorff's spatial and space-time scan statistics [32], and genetic algorithms for spatial [33] and space-time clustering [34].

GIS analysis supported with novel clustering algorithms have become valuable tools in environmental health studies for studying the spatial distribution of environmental contaminants and potential risks associated with diseases [35-38]. For example, SOM was employed to evaluate dioxin patterns in mother milk and dietary habits from various countries and identify contributing dietary factors in different countries [36]. Methods, such as spatial scan statistic or boundary analysis have been applied to various types of cancers to analyze the impact of pesticide use [38] or air toxicity [35]. However, there is little focus on the spatial relationship between increased breast cancer incidence and background exposure to dioxin in soils. In this study, we aimed at (1) evaluating dioxin contamination in the study area and (2) investigating the hypothesis that dioxin-contaminated areas are spatially associated with high breast cancer incidence rates. Answers to the first objective provided information to understand the extents and severity of dioxin contamination and the contributing factors. Areas with high levels of dioxins can be targeted for cleanup with higher priority. Answers to the second objective would be important in targeting areas identified as having high incidences of breast cancer for heightened surveillance and education, as well as formulating new hypotheses for further research.

## Methods

### Study area, population, and major river systems

The study area (Figure 1) consists of Midland, Saginaw, and Bay Counties. It has 38 ZIP codes with a population of over 400,000 [39,40]. Midland, Saginaw, and Bay Cities are three densely populated regions. The study area has several industries, notably the Dow's Midland plant, making significant contributions to economic growth in this region.

The Tittabawassee and Saginaw Rivers are two major river systems. The Tittabawassee River extends southeast from the city of Midland to the confluence of the Tittabawassee and Saginaw Rivers. The Saginaw River flows east into the Saginaw Bay on Lake Huron. Land use in the Tittabawassee River floodplain splits among residential, agricultural, public parks, and protected areas, i.e., Shiawassee National Wildlife Refuge (NWR). The Tittabawassee River has seen frequent floods resulting from rain and/or snow melt. The 1986 fall flood was classified once every 100–500 years. In 2004 spring, another extensive flood struck the area. Some of the flooded areas are currently used as private backyards or public parks.

### Breast cancer data

Data pertaining to invasive female breast cancer cases (n = 4,604) diagnosed for 1985 through 2002 were obtained from the MDCH. The cancer registry in the MDCH maintains the highest standards for data quality and completeness. It was complied under the National Cancer Institute's Surveillance, Epidemiology, and End Results (SEER) Program. A breast cancer case was defined as a person with any newly diagnosed cancer with a behavior code of "3" (malignant primary site) and a site group of "12" (breast) as classified according to the International Classification for Diseases 10^th ^revision (ICD-10). Each record represents a newly diagnosed breast cancer case assigned to the patient's residence at diagnosis. Each case includes information on patient's gender, ZIP code of a patient's residence, year of diagnosis, primary site, stage at diagnosis, and age group (Table 1). To protect privacy, the MDCH registers patients at ZIP code level rather than individual physical residences and only publically reports ZIP codes with more than 5,000 people. Of the 38 ZIP codes in the study area, only 22 ZIP codes have breast cancer data with 90.8% (378,831/417,423) of the study population living there. The lack of data for the remaining 16 ZIP codes means that we were not able to fully investigate them. And it is still unclear whether there were no diagnosed or unreported cases, or simply the MDCH concealed it to protect patient privacy.

**Table 1:** Number of cases for female breast cancer, by age-group

Age (in years)	Cases
≤14^a^	1

15 – 44	572

45 – 64	1,833

65 – 74	1,126

≥75	1,071

Unknown^b^	1

Total cases	4,604

^a ^The record was removed from the analysis as merging into other groups may bias the underlying at-risk background population in other groups.

^b ^The record was removed from the analysis because of missing age.

### Soil dioxin data

The Michigan Department of Environmental Quality (MDEQ) provided the soil dioxin database consisting of 532 records (Figure 1) collected mainly from the city of Midland and the Tittabawassee River floodplain between 1995 and 2003, respectively. In the database, each record has a unique identification number, the coordinates of the sample location, starting depth, ending depth, dioxin concentration in parts per trillion (ppt TEQ) toxic equivalents, and the year of the sample. The TEQ values are given on a dry weight basis and it is only for PCDDs and PCDFs (not PCBs). Most samples were collected in surface soil from 0–1, 0–2 or 0–3 inches. Besides the topsoil samples, additional samples were collected below surface from 3–6, 12–15, 16–24, 36–48 or 48–60 inches downstream of the Tittabawassee River. This database is quite comprehensive and includes soil TEQ concentrations from three counties collected over various sampling efforts between 1995 and 2003 by the MDEQ, EPA, Dow, and U.S. Army Corps of Engineers (USACE). In addition, it takes into account the total toxicity of all toxic dioxin congeners by using the complete toxic equivalent approach, not only considering TCDD.

Additional geographic information used includes ZIP code boundary, county boundary, rivers, roadways, flood frequency, and census data in both 1990 and 2000. They were obtained from the MDEQ, the Michigan Center for Geographic Information (MCGI) and U.S. Census Bureau. The flood frequency is classified as 1-, 2-, 5-, 10-, 50-, 100-, and 500-year according to floodway data published by the U.S. Federal Emergency Management Agency (FEMA). For example, a 5-year floodplain refers to an area adjacent to a river that is expected to flood once every 5 years.

### Data analysis

We used a variety of methods to process and analyze the data. These methods include (1) evaluation of soil dioxin contamination by using descriptive statistics and the SOM algorithm; (2) evaluation of the association between breast cancer rates and the ZIP codes by estimating the odds ratio and their corresponding 95% confidence intervals; and (3) cluster detection using Kulldorff's spatial and space-time scan statistics, and genetic algorithms for spatial and space-time clustering.

The SOM is an unsupervised data visualization and classification technique that reduces high-dimensional data to lower, usually 1 or 2, dimensions [31]. Compared to variance-covariance matrix and multi-dimensional scaling, the SOM allows one to visually figure out the number of clusters, the classification of different values of each variable, and relations between variables. The SOM consists of processing elements (neurons). Each neuron is represented by a *d*-dimensional weight vector, where *d *is equal to the dimension of the input vector. In our case, four input vectors are dioxin level (Dioxin Level), distance from a sampling site to the river (Distance to River), flood frequency of a sampling site (Flood Frequency), and start depth where a sample collection begins (Start Depth). Neurons are connected through a neighborhood function (*f*), e.g. a Gaussian function defined by $f(t)=e^{-d^{2}/2\sigma_{t}^{2}}$, where *d *is the Euclidean distance between two neurons and *σ*_*t *_is the neighborhood radius at time *t*. Hidden layers (*n*) act as intermediate layers between the input vector layer and output layer. The SOM then uses the input vectors to update neurons in the hidden layer to generate the next hidden layer or output layer. The update is conducted using a learning rule to train neurons, e.g. *n*_*i*_(*t *+ 1) = *n*_*i*_(*t*) + *α *(*t*)*f*_*i*_(*t*) [*x*(*t*) - *n*_*i*_(*t*)], where *i *is the *i*^*th *^neuron; *x*(*t*) is an input vector from the input data set at time *t*; and *α*(*t*) is the learning rate at time *t*. The aim of the update process is to make neurons more like the input vector; the end result is that the neurons on the map become ordered and neighboring neurons are similar. The output map consists of the U-matrix and component planes. Neurons in the U-matrix with small values represent clusters in the input data and large values represent gaps. Each component represents an attribute and its classification from the input data. The neuron in a certain position in one map corresponds to the same neuron in other maps. By reading several component planes and their color legends together, it is easy to examine the correlations between different attributes. See reference [41] for a detailed SOM description. We implemented the SOM model using SOM Toolbox [41] and MatLab 7.1 (The MathWorks, Inc, Natrick, Massachusetts). The SOM model may be viewed as non-linear extensions of standard regression models in the sense that it performs various non-linear mappings between the variables in the input, hidden, and output layers [42]. The distance in feet from a sample site to the river and the flood frequency of each sampling site were obtained using ArcGIS 9.2 (Environmental Systems Research Institute, Inc, Redlands, California).

The statistical analysis included the estimation of odds ratio and 95% confidence intervals adjusted for age at a significance level of *p *≤ 0.05 using SAS 9.1 (SAS Institute, Inc, Cary, North Carolina) and Microsoft Excel (Microsoft, Inc, Redmond, Washington). Our null hypothesis was that high breast cancer incidence rates are randomly distributed in the 22 ZIP codes. The alternative hypothesis was the breast cancer rates increase when the geographic locations are close to the dioxin contamination areas. We used ZIP code 48883, an area located upstream of the river (Figure 1) as the reference for comparison. Given that the levels of dioxins in this area are close to background levels across Michigan as reported by previous studies [1,3,4,8], the population was assumed to be unexposed to dioxin. The female populations in this ZIP code in both 1990 and 2000 are close to the average female population per ZIP code in the study area. To test how sensitive the result would be, we conducted a comparative analysis with remote ZIP codes 48618, 48657, 48650, 48616, and 48655 as alternative references. Residents living in these ZIP codes were assumed to be farther away from the contaminated area and have less chance of being exposed to dioxins.

The incident rates were only adjusted for age as a covariate because patient's race and other socio-economic status were not provided in the breast cancer database. Census data were linked to cases based on the ZIP code of residence at the time of diagnosis. We completed this task using ArcGIS 9.2 to join ZIP code boundary data with breast cancer and census data. All cases were matched with respective female demographics and their corresponding age groups. For input data to the space-time scan and genetic algorithm models [32,33], we preprocessed data and projected populations to obtain values between 1990 and 2000 using linear regression. For these models, we assumed that populations before 1990 and after 2000 were the same as the official U.S. census count for the two periods 1990 and 2000.

The spatial techniques used to detect spatial clusters of breast cancer incidence include Kulldorff's spatial and space-time scan statistics [32], and the genetic algorithms for spatial [33] and space-time clustering [34]. We first used the spatial scan statistic and genetic algorithm for spatial clustering to explore whether spatial clusters of breast cancer exist in our study area. We then used the space-time scan statistic and the genetic algorithm for space-time clustering to locate clusters in space-time. Kulldorff's spatial and space-time scan statistics were applied to test the null hypothesis (at *α *= 0.05) that no clusters of increased breast cancer incidences exist on the basis of 999 Monte Carlo replications. The GIS mapping tool was employed to review the resulting spatial clusters of breast cancer and any potential risks in locations suspected to be contaminated by dioxins.

Kulldorff's spatial and space-time scan statistics, built in SatScan 7.0 (developed jointly by Kulldorff M., Boston, Massachusetts and Information Management Services, Inc, Silver Spring, Maryland), are popular cluster detection tests appropriate for handling aggregated spatial data. The spatial scan statistic imposes a circular or elliptic search window on the study area. The space-time statistic uses a conic search window where the base is circular or elliptic and the height corresponds to the time interval. The cases within a search window represent a potential cluster. The search window then varies in size in each data point successively. Because the number of events in an area at one time follows Poisson distribution, the expected number of events within a search window is proportional to at-risk background population size when there are no covariates. Under the Poisson assumption, the method calculates the likelihood function for all windows. The one with the maximum likelihood represents the most likely cluster, and this cluster is least likely to have occurred by chance [32]. The method then conducts the maximum likelihood ratio test statistic and obtains the *P*-value through Monte Carlo hypothesis testing [43]. The test result shows whether the number of case patients within the search window with maximum likelihood constitutes the disease cluster and whether this disease cluster is statistically significant (at *α *= 0.05). The scan statistics themselves are advantageous and guarantee to find clusters if they exist; however, the SatScan 7.0 software restricts the ratios of the longest to the shortest axis of an ellipse to 1.5, 2, 3, 4 or 5 and limits the number of directions as 4, 6, 9, 12, and 15. Given that shapes and directions of clusters are usually unknown before analysis, such restrictions may include too many at-risk background populations. Therefore, a method that can "relax" these assumptions is highly desirable to validate the results.

Genetic algorithms for spatial clustering [33] and for space-time clustering [34] were employed to explore spatial patterns of breast cancer incidences and further confirm the results from Kulldorff's methods. Compared with Kulldorff's methods, the genetic algorithms do not restrict the ratios of the longest axis to the shortest axis and allow arbitrary directions of ellipses. Therefore they provide finer delineations of clusters without including unnecessary at-risk background population, thus effectively detecting long and narrow clusters. Genetic algorithms are randomized search techniques simulating the principle of survival of the fittest. They are effective in cluster detection [44] by producing near-optimal solutions to search problems. Each genetic algorithm consists of an initialization step, a pre-specified number of iterative generations, and three genetic operators (namely, reproduction, crossover, and mutation). The initialization step randomly generates a set of strings (chromosomes). This set of strings is called the population. In our case, each string is an ellipse with five parameters (*x*, *y*, *a*, *b*, *θ*), where *x *and *y *are the centroid coordinate of an ellipse; *a *and *b *are semi-major and semi-minor axes respectively; *θ *is a positive real number representing the orientation angle with a range from 0 to 180°. Cases within an ellipse represent a potential cluster. After the initialization step, there is an iteration of generations. In each generation, three genetic operators will run on the population. The fitness value of each string is first calculated according to a fitness function, e.g. $c-C\times \frac{p}{P}$, where *c *and *p *are the actual number of disease cases and population in an ellipse; and *C *and *P *are the total number of cases and population in the study area respectively. A string (ellipse) will be exported into a cluster list if its fitness value is larger than 0 under the Poisson assumption. The reproduction operator selects a set of strings that have higher fitness values. These selected strings become strings (children) in the next generation. Crossover then chooses a proportion (crossover rate) of the children strings and mates each pair on a randomly located position. In our case, a random integer in (1, 5) generated for each pair, e.g., 5 allows the two chosen ellipses to exchange their directions and become two new ellipses. Mutation selects bits of the mated strings with a probability (mutation rate) and changes the value on a randomly generated position on each string. In our case, an ellipse may have its position, shape, or direction mutated. A number of randomly generated strings will then be placed into the next generation to maintain the population size. The algorithm keeps updating the population for the number of iterations, aiming at preserving ellipses with higher fitness values while searching in new areas. The genetic algorithm for space-time clustering uses elliptic cylinders as strings with an elliptic base and height corresponding to time interval within a study period. Each string has 7 parameters (*x*, *y*, *a*, *b*, *θ*, *T*_*s*_, *T*_*e*_), where *T*_*s *_and *T*_*e *_are starting and ending time respectively. Similar to SatScan, the genetic algorithms can adjust for covariate by comparing the observed number of case patients in a category with the corresponding underlying at-risk background population. We implemented the two clustering algorithms based on the genetic algorithm toolbox 1.2 [45]. The performance evaluation shows that the methods are accurate and reliable. A detailed description of the algorithms is presented in [33,34].

### Assumptions for exposure assessment of breast cancer incidences and soil dioxin contamination

It was assumed that those who live in or close to ZIP codes where dioxin levels in soil exceed the Michigan soil generic residential direct contact criterion (RDCC) for dioxin of 90 ppt TEQ were exposed to dioxin, and those living farther away were assumed to be unexposed. We used the ZIP code of residence at diagnosis as an indicator of exposure and did not account for migration between ZIP codes and cancer latency. This is a typical assumption in other ecological studies [37,38,46,47] because of privacy concerns and limited availability of personal information. Although ZIP codes are arbitrary units of analysis in terms of contamination and potential health outcomes, it is convenient for public health agencies to register patients at this level so as to protect patient privacy [35,46]. In addition, we did not account for migration because of low mobility among the study population as was reported in several surveys – one survey [48] indicated that sixty-six percent of respondents in the Tittabawassee River floodplain have lived at their current residence for more than 10 years (Table 2); another survey [8] reported that residents in Midland and Saginaw Counties have lived there for an average of almost 40 years; and more importantly, people in the Tittabawassee River floodplain have the longest length of residence of 45 years.

**Table 2:** Description of length of stay at current residence

Length of stay at current residence	Percent (%)
Less than 1 year	0.5

1 to 5 years	12.9

6 to 10 years	20.4

11 to 20 years	22.0

21 to 30 years	18.8

More than 30 years	25.2

No Response	0.5

Note: About sixty-six percent of respondents lived at the current residence for more than 10 years (Source: [48]).

We further assumed that the dioxin data from 1995 to 2003 represents dioxin levels in the preceding period when causative exposure may have occurred. Dioxin samples were collected from 1995 through 2003, and cancer cases ranged from 1985 through 2002. One critique remains, as dioxin in 2000 could not have caused cancers developed in 1985. Jacquez and Greiling [35] argued that because of the latency in the development of cancer, it would not even be plausible to say that contamination in 1998 could explain only 1999 diagnoses. The year of 1995 is when a comprehensive dioxin sampling was available for the study area; however, previous smaller samples of dioxin data were collected way back in 1983 [3]. Taking into account dioxins' long half-lives and resistance to biodegradation, we assumed dioxin data from 1995 to 2003 adequately represent dioxin levels in soils prior to 1995. Although this assumption is not ideal, it is reasonable because occurrence of elevated concentrations of PCDD/Fs in sediments at depths below 60" in the river indicates that the contamination is occurring historically [7], mainly due to the operation of fairly inefficient incinerators in the Dow's plant since 1940s [3]. The moderation of the facilities (99.9999% destruction of dioxins) in 2000 resulted in significant reduced emissions [3]; however, the contamination in soils has not received major remediation yet [1,3,4]. To account for the effects of long cancer latency, we used the cancer data starting from 1985 rather than using recent data only.

## Results

**Figure 2:**
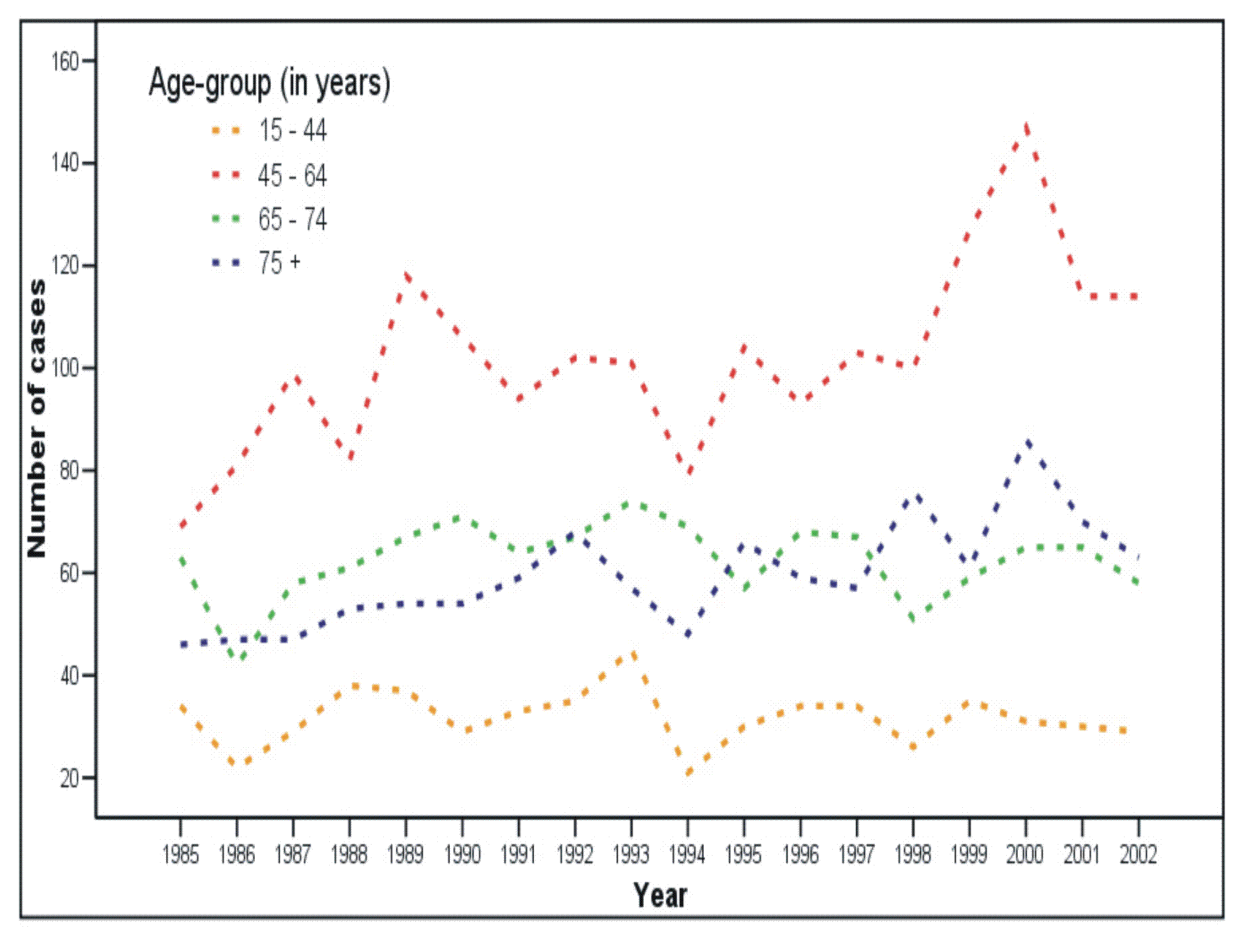
**Number of breast cancer cases in Midland, Saginaw, and Bay Counties, Michigan**. Number of cases for female breast cancer by year and age-group, Midland, Saginaw, and Bay Counties, 1985 to 2002.

From 1985 to 2002, there was an increasing trend in the number of breast cancer cases in females between 45 and 64 years old in Midland, Saginaw, and Bay Counties (Figure 2) with an APC of 0.43, which is slightly higher than the national trend (0.4) during the approximately same period [24]. These females are apparently overrepresented and have the highest risk in all age groups. Cases among females aged over 65 years remained relatively stable during the study period, while females aged between 15 and 44 had the lowest risk.

**Figure 3:**
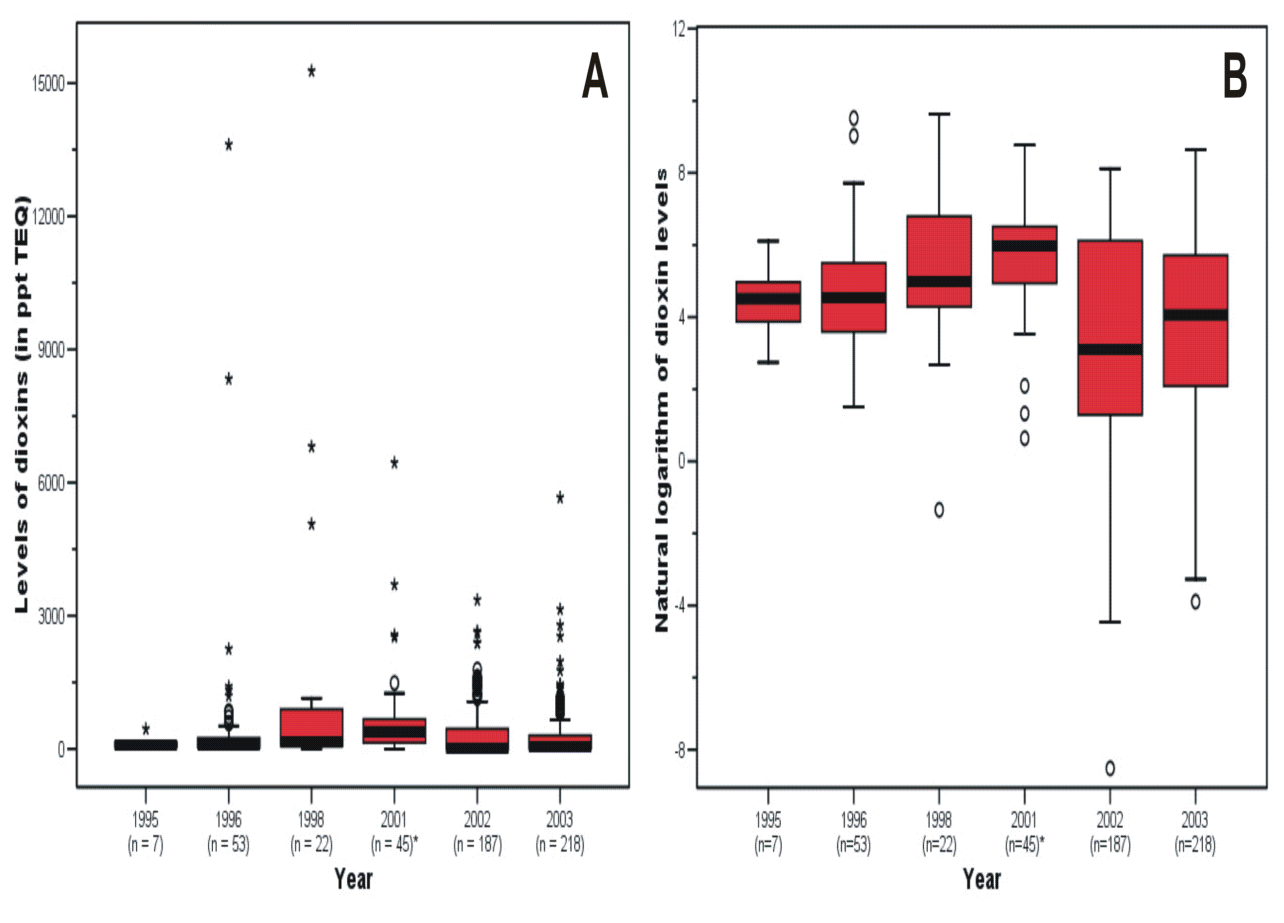
**Box plots of dioxin concentrations in the city of Midland and the Tittabawassee River floodplain, Michigan**. Dioxin concentrations by year in the city of Midland and the Tittabawassee River floodplain, Michigan, 1995 to 2003. Logarithm transformation of the soil generic RDCC for dioxins (90 ppt TEQ) is 4.5. Samples in 2000 and 2001 were merged because there were only two samples from 2000.

The statistical evaluation of dioxin data suggests the levels of dioxins vary widely in the area (Table 3). If we use 90 ppt TEQ (the soil generic RDCC for dioxin) and 1,000 ppt TEQ (the action level for dioxin suggested by the U.S. Centers for Disease Control and Prevention and the U.S. Agency for Toxic Substances and Disease Registry) as benchmarks, the average for most samples exceed the two standards. In vertical extent, dioxin contamination occurs mainly in shallow layers of soil, especially in 0–6 inches. However, six samples at 15–36 inches with 1,620 ppt TEQ show that dioxin contamination exists in deeper layers as well. In geographical extent, most samples in Midland exceeded 90 ppt TEQ. Along the Tittabawassee River, samples within the 100-year floodplain range up to 6450 ppt TEQ. The levels of dioxins in samples within or beyond 500-year floodplain were below 90 ppt TEQ. Three samples collected by USACE along the Saginaw River exceeded 90 ppt TEQ, which is consistent with a previous study [7]. In temporal extent, the box plots of dioxins plotted using original values (Figure 3a) and natural logarithm transformation (Figure 3b) provide insighful variations of dioxin contamination during the study period. For example, dioxin concentrations in most samples remained consistently above the criterion of 90 ppt TEQ from 1995 to 2003. Outliers with extremely high concentrations (Figure 3a) were apparent, especially in 1996, 1998, and 2003. The variations within and between years are more pronounced by using logarithm transformation. The 25^th ^and 75^th ^percentiles show persistent dioxin contamination in the sampling period.

**Table 3:** Frequency distribution of the levels (ppt TEQ) for dioxins in soil classified by depth and location

Categories	Sample size	Mean	Min	Max	Stdev	25^th ^P	75^th ^P	Med
**Depth (in inches)**								

0 – 3	307	500	0	15,300	1,440	15	442	83

3 – 6	112	350	0	2,790	590	5.56	525	56.3

6 – 15	102	355	0	5,660	799	3.14	356	44.3

15 – 24	3	576	6.9	1,620	909	6.9	1,620	97.5

24 – 36	3	56.9	13.9	133	65.9	13.9	133	23.9

36 – 48	3	24.1	1.12	64	34.6	1.12	64	7.34

48 – 60	2	6.77	1.42	12.1	7.57	1.42	12.1	6.77

**Locations**								

Midland	53	1,140	0	15,300	3,040	69.6	111	727

Floodplain								

1-year	191	564	0	5,660	725	62.1	923	308

2-year	80	364	0.17	3,140	632	30.4	438	99.3

5-year	29	39.8	0.18	294	74.2	5.63	32.5	16

10-year	32	34.6	0	244	62.2	3.53	41.2	7.17

50-year	44	206	0	5,070	792	2.23	34.1	8.74

100-year	43	481	0	6,450	1,130	1.1	531	26.4

500-year	15	13.7	0.23	69.7	20.1	3.45	11.6	4.5

Outside	35	13.9	0	76.8	20.3	1.8	22.4	4.55

Saginaw	10	96.3	0.73	450	136	9.81	142	50.4

Total	532							

^a^Stdev, standard deviation

**Figure 4:**
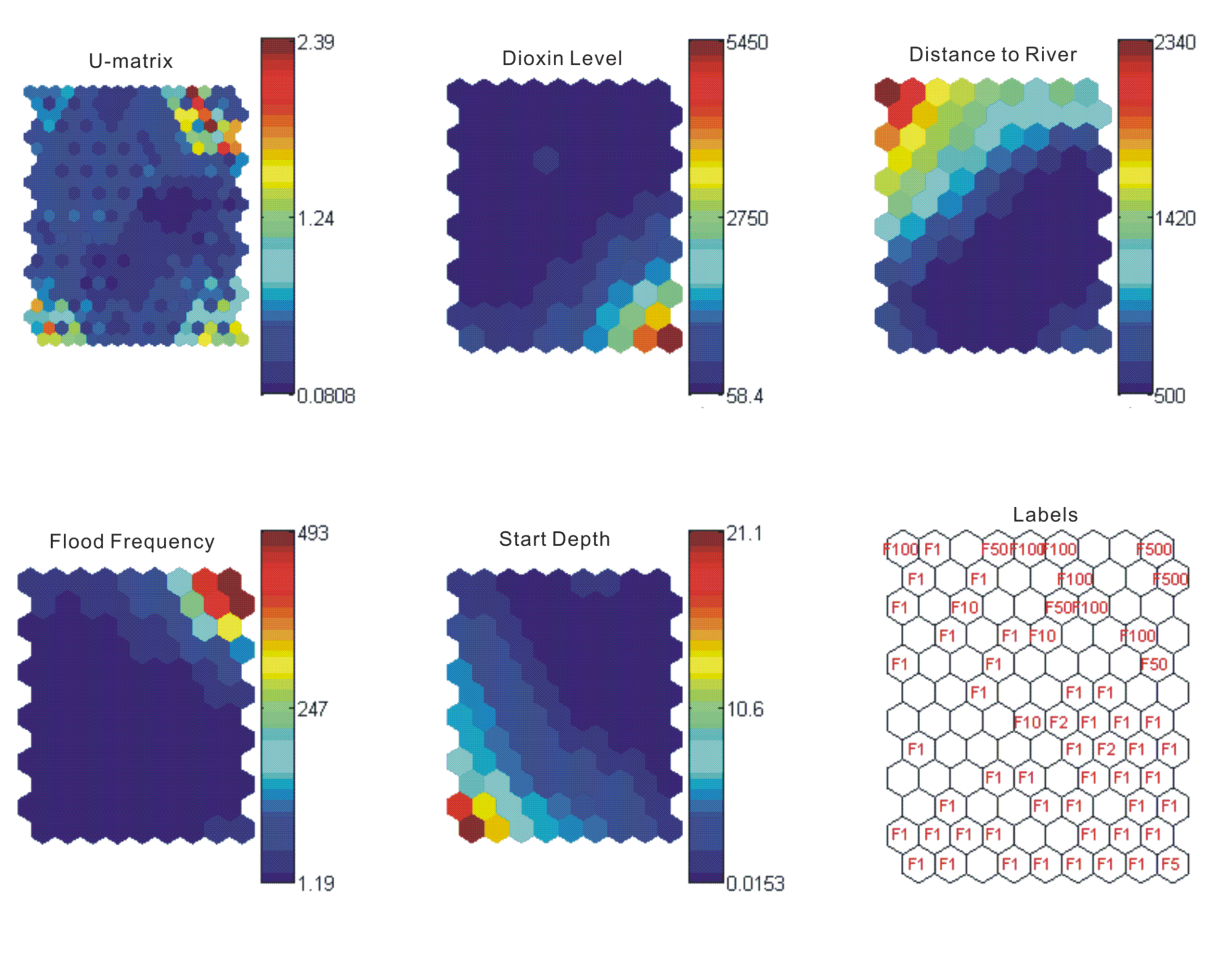
**U-matrix, four component planes, and map unit labels, from the SOM on the dioxin database**. The U-matrix shows dioxin levels vary across the study area. Comparisons of the four components and labels indicate that soil dioxin contamination is limited within the 100-year flood frequency area. Very high levels of dioxin are clustered within the 10-year flood frequency area. Soil deeper than 16" below the ground is contaminated by dioxin in some areas. Dioxin contamination is alleviative in areas farther away from the river, but not linearly.

The SOM plots show key characteristics of dioxin contamination in the floodplain (Figure 4). The U-matrix indicates that high levels of dioxin are clustered (dark blue neurons in the U-matrix) in some geographical locations. A comparison of neuron values of the four component planes reveals some interesting correlations between dioxin levels and other input variables. For instance, the corresponding neurons on the bottom right corner of the four component planes illustrates a correlation in high dioxin levels (Dioxin Level) (1) with closeness to the river (Distance to River); (2) with highly frequent flooding events (Flood Frequency); and (3) with shallow soil layers (Start Depth). It indicates a 5-year flood frequent area (Labels). Two observations can be made from the comparison between Dioxin Level and Labels. First, dioxin contamination resides in the 100-year floodplain. Very high levels of dioxin exist within the 10-year floodplains. Areas with flood frequency from 50- to 100-year have similar intensities of dioxin contamination, identified as lower levels of dioxins. Second, areas with flood frequency from 100- to 500-year have dioxin levels around 58.4 ppt TEQ and can be classified as uncontaminated areas. The comparison between Labels and Start Depth indicate that very high levels of dioxin exist mostly in shallow soil layers. But in soils deeper than 16" below the ground in some areas, dioxin levels exceeded 90 ppt TEQ, this may be in part due to flood plain sediments being used as infill in housing projects. The comparisons between dioxin level and Distance to River and Flood Frequency indicate that flood frequency rather sampling distance to the river can better estimate levels of soil dioxin. Higher levels of dioxins usually reside in areas closer to the river that experience more floods. However, the neurons at bottom right corner of three component planes suggest high levels of soil dioxins in highly flooding areas and low levels as you move farther away from the river. This demonstrates that significant local elevation change may occur and thus prohibits the direct use of distance to the river as an indicator of dioxin contamination.

The distribution of breast cancer incidence rates by age and ZIP code between 1985 and 2002 are presented (Table 4). Female patients over 45 years old had two times a higher risk of developing breast cancer than females aged 15 to 44. Most of the patients (88%) were older than 45 years. Female patients between 45 and 64 years old had the highest risk (39.8%) in comparison to other age groups. However, those were between 65 and 74 years old had the highest breast cancer incidence rates (469 per 100,000 females). Geographically, cities of Midland, Saginaw, and the immediate vicinity (ZIP codes 48640, 48601, and 48603) had the highest breast cancer incidence rates. Locations that are in close proximity to dioxin-contaminated areas have higher breast cancer incidence rates than those located farther away after adjusting for age. For example, the highest risk of breast cancer was observed west of Midland (48640), in the confluence area of the Tittabawassee and Saginaw Rivers (48603). In addition, the cities of Midland and Saginaw (48640 and 48602) and southwest Midland (48880) have statistically higher breast cancer incidence rates (*p *≤ 0.05). Overall, females living in these areas had a statistically significant risk that increased from one-third to two-thirds in comparison with females living in reference ZIP code 48883. The comparative analysis shows a statistically significant increase of the rate of breast cancer (*p *≤ 0.05) in Midland (48640), Saginaw (48602 and 48603), and southwest Midland (48880) in comparison to ZIP code 48616. When other ZIP codes (48618, 48657, 48650 and 48655) are used as references, only Midland (48640) consistently presents a statistically significant increase of the rate of breast cancer. The rates increase from 36 to 64 percent. The comparisons suggest a decrease of the rate of breast cancer on the east side of Midland (48642) reducing from 46 to 63 percent.

**Table 4:** Distribution of age and ZIP code for breast cancer cases, 1985–-2002

	% Diagnosed Breast Cancer	Breast Cancer Rates (per 100,000)	Odds Ratio	95% CI^a^	P-value
Age (years)					

15–44	12.47	39	1		

45–64	39.81	227	5.81^b^	1.65, 1.86	<0.0001

65–74	24.46	469	11.94^b^	2.37, 2.60	<0.0001

≥75	23.26	459	2.45^b^	2.34, 2.57	<0.0001

ZIP code					

48883	1.30	90	1		

48415	2.35	123	1.28	-0.11, 0.60	0.1699

48457	1.76	108	1.13	-0.25, 0.50	0.5163

48601	11.29	112	1.25	-0.08, 0.52	0.1526

48602	8.93	133	1.39^b^	0.03, 0.64	0.0309

48603	13.79	171	1.34	-0.01, 0.58	0.0579

48604	3.17	133	1.34	-0.04, 0.63	0.0877

48611	1.46	126	1.22	-0.19, 0.59	0.3160

48616	1.63	110	1.01	-0.37, 0.39	0.9657

48618	1.41	139	1.35	-0.10, 0.69	0.1407

48623	2.32	105	1.15	-0.22, 0.49	0.4546

48626	1.30	112	1.13	-0.28, 0.52	0.5451

48640	12.08	184	1.86^b^	0.32, 0.92	<.0001

48642	3.82	60	0.63^b^	-0.80, -0.14	0.0047

48650	1.89	127	1.2	-0.19, 0.55	0.3430

48655	1.65	126	1.26	-0.15, 0.61	0.2408

48657	2.17	137	1.35	-0.06, 0.66	0.0982

48706	11.40	138	1.2	-0.12, 0.47	0.2509

48708	7.67	131	1.25	-0.08, 0.53	0.1539

48732	3.58	145	1.22	-0.13, 0.53	0.2356

48734	2.80	183	1.3	-0.09, 0.60	0.1438

48880	2.22	171	1.88^b^	0.27, 0.98	0.0006

Note: ZIP codes 48640 and 48602 are located in the city of Midland and the confluence area of the Tittabawassee and Saginaw Rivers, respectively. If a simple Bonferroni's adjustment is applied (0.05/21), *p *< 0.0024 can be used to judge significance. This criterion makes ZIP codes 48640 and 48880 more significant than others.

^a ^CI, confidence interval.

^b ^statistically significant at the 5% significance level.

**Figure 5:**
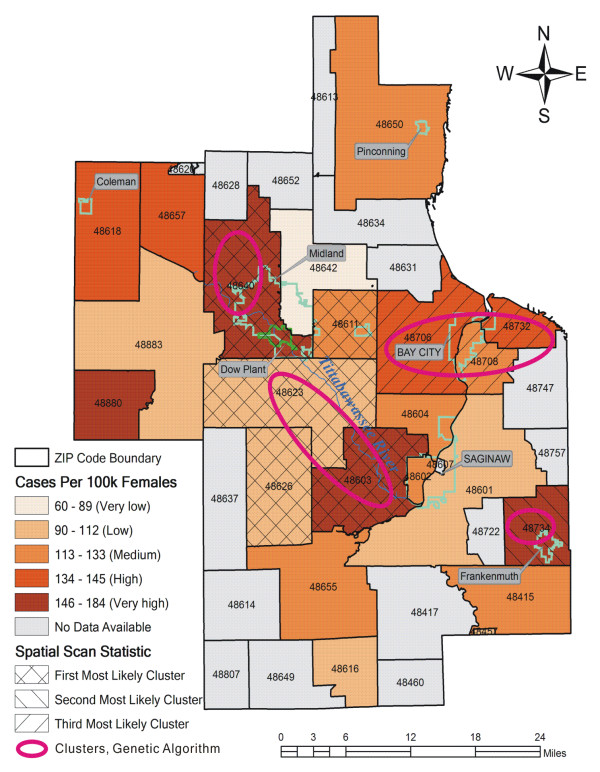
**Breast cancer cases per 100,000 females, and purely spatial clusters detected by the two methods**. Purely spatial clusters detected by Kulldorff's spatial scan statistic (shaded areas) and the genetic algorithm for spatial clustering (ellipses).

The map shows the spatial distribution of breast cancer incidence rates per 100,000 females and spatial clusters after adjusting for age (Figure 5). In this map, high breast cancer rates are located in close proximity to the suspected areas contaminated by dioxins; as you move farther away from these contaminated areas lower rates become more evident. The spatial scan model returned three most likely clusters – illustrated in shaded patterns on the map – with the first most likely cluster located in or near to the contaminated areas (*p *= 0.001). This cluster has 31% of the total breast cancer cases. It consists of ZIP codes 48640, 48603, 48623, 48626, and 48611. The second most likely cluster (48734) was located farther away from the contaminated areas (*p *= 0.014). The third cluster was centered on Bay city (48708, 48732, and 48706), but not statistically significant at the 95% confidence level (*p *= 0.906). Using the genetic algorithm, we observed four spatial clusters of breast cancer incidences illustrated by ellipses. Among the four clusters, two of them are close to the contaminated areas.

**Figure 6:**
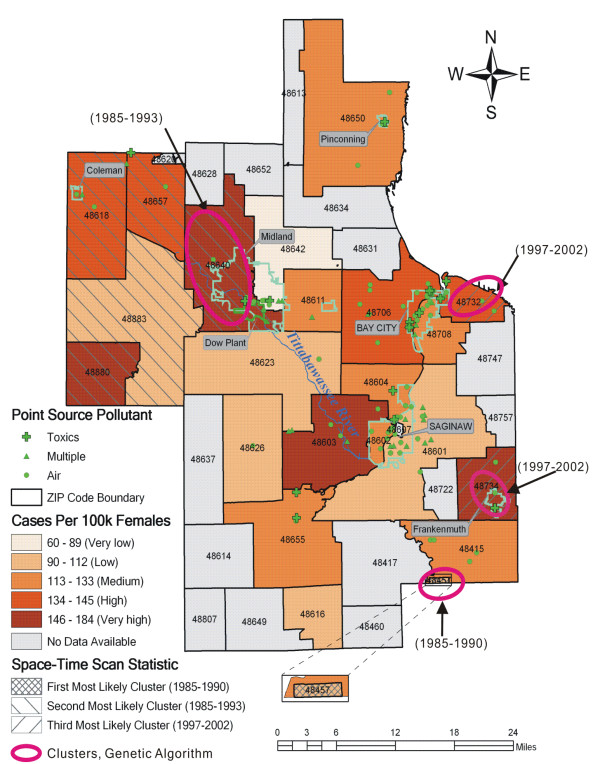
**Breast cancer cases per 100,000 females, space-time clusters, and point source pollution sites**. Space-time clusters detected by Kulldorff's space-time scan statistic (shaded areas) and the genetic algorithm for space-time clustering (ellipses).

Using the space-time clustering techniques, we detected the breast cancer clusters in space-time after adjusting for age (Figure 6). The space-time scan model returned three clusters; with the first most likely cluster (ZIP code 48457 from 1985 to 1990) located away from the contaminated area (*p *= 0.001). It has 23 cases with at-risk background population of 1052. The second one (1985–1993) is a super cluster located west of and in the city of Midland (*p *= 0.001). It has 445 cases and 16735 at-risk female population. The third (1997–2002) is located in the Eastern Bay city and Frankenmuth (*p *= 0.001). It has 106 cases and 4142 females at risk in the same period of time. The genetic algorithm for space-time clustering returned four clusters delineated by ellipses (Figure 6): ZIP code 48640 (1985–1993) in Midland as a single cluster, ZIP code 48457 (1985–1990), and ZIP codes 48732 (1997–2002) close to Bay city and Frankenmuth located in ZIP code 48734 (1996–2002).

## Discussion

In this study, we evaluated levels of dioxin in soils and analyzed spatial variations in the incidence of breast cancer. There are four major findings from this study: (1) dioxin contamination sites include the city of Midland and the Tittabawassee River 100-year floodplain. Very high levels of dioxins are limited in the areas with 10-year flood frequency; (2) the number of breast cancer cases increased from 1985 to 2002 among females aged between 45 and 64 years and they had the highest risk; (3) rather than randomly distributed in the study area, ZIP codes where high breast cancer rates exist are clustered in or near to the contaminated areas after adjustment for age; and (4) living on or close to the contaminated areas is spatially associated with the increased incidences of breast cancer. Findings in this study are consistent with findings from previous studies [1,3,4,8,16,18,19,29,49,50].

Previous epidemiological studies have found increased breast cancer incidence [17-19] and mortality [20,21] in females exposed to dioxins. Yet epidemiological studies are vulnerable given insufficient sample sizes [14,19]. Spatial techniques in cancer studies have contributed to the understanding of disease etiology and the impact of contaminants [35,37,38]. However, little attention has been paid to using spatial techniques to evaluate dioxin contamination and to analyze its spatial association with breast cancer rates. Our study takes advantage of publicly available historical data, GIS, and spatial and statistical analysis techniques. Publicly available historical data on breast cancer provide an opportunity to quickly understand the spatial variation of the disease. The final spatial models presented for this study using maps illustrate a nonhomogenous distribution of breast cancer incidence rates and potential risks associated with soil dioxin contamination among women in three counties.

Findings in this study gave some interesting insights about the characteristics of dioxin contamination. The most important insight was that contaminated areas were predominantly the city of Midland and the Tittabawassee River 100-year floodplain. Air deposition from historical operations at the Dow and soil relocation activities may explain the presence of very high levels of dioxins in Midland [3]. Flood may be a contributing factor that continuously sweep and redeposit contaminated soil and sediments in the floodplain [7,8]. Sudden elevation change, soil relocation activities, or physical barriers to floods may explain the low levels of dioxins in highly flooding areas. The small sample size in deeper soil layers and along the Saginaw River warrants additional samples to determine if the distribution of dioxin is consistent. We settled for the SOM technique partly due to the following reasons. The dioxin data had significant number of outliers with extremely high TEQ values even after log transformation of the data, thus remaining outliers and nonhomogeneous variations between groups made classical statistical methods less reliable. Our approach complements Goovaerts's recently modified geostatistical method that was used to analyze soil dioxin distribution in the vicinity of an incinerator in Midland [3,4].

Preliminary statistical analysis suggests that there is a strong association between elevated levels of breast cancer incidence and aging, particularly among females residing in the city of Midland or near areas contaminated with high dioxins levels. In fact, breast cancer incidence rates increase significantly (*α *= 0.05) as women get older, which is consistent with findings from previous studies [22,38,49,51]. In addition, the city of Midland, where the high levels of dioxins exist, had a statistically significant (*α *= 0.05) increased rate of breast cancer. The statistical significance was confidently reaffirmed after conducting a comparative analysis using five different remote ZIP codes serving as references, suggesting there are important factors contributing to the high incidence of breast cancer in Midland.

Findings from this study reveal that there are elevated levels of breast cancer incidence in areas or near areas contaminated by dioxins. Residents living in or near to these contaminated areas are more likely to visit these areas; therefore, they are more likely to have been exposed to dioxins than residents living far away. Findings from the Dioxin Exposure Study [8] may support this argument. Long-term exposure due to air deposition of high concentrations of dioxins from inefficient incinerators in Midland presents a significant health hazard to local residents [3]. Other pathways may also expose local residents to high risks, e.g., direct soil and household dust contact, using contaminated sediments infill material in housing projects, eating fish and game from the contaminated area, doing water-related activities in the contaminated area, and working at the Dow [8]. Findings in the study [8] report that forty-six percent of people living on the floodplain have swum, picnicked, hiked, boated, and participated in other recreational activities in and around the Tittabawassee River, compared to 31% in the near floodplain, and 21% in other areas from Midland and Saginaw Counties. The same study indicates that people who live on the floodplain are the most likely to have fished in the river during their lifetime.

The cluster analysis provided further evidence of spatial association between greatly elevated levels of breast cancer incidence rates and soil dioxin contamination. The results from Kulldorff's methods and the genetic algorithms are consistent with the findings from the statistical analysis above. The city of Midland was found to have a breast cancer cluster in both space and space-time. The large female population in Midland (13,221 in 1990 and 16,796 in 2000) suggests this cluster occurred less likely by chance. The detection of clusters in ZIP codes 48611, 48623 and 48626 (Figure 5) is a false positive, since these ZIP codes have much lower rates and percent of breast cancer than the other ones (see Table 3). This is a common shortcoming of the clustering algorithms in use as they rely on minimum population size to detect high rates. The interpretation of clusters in Bay city (Figures 5 and 6) takes caution. Although these clusters are far away from Midland and the Tittabawassee River, in one recent study [7] it was reported that sediment and floodplain soils of the Saginaw River, where these clusters are, are considerably contaminated with high levels of dioxins similar to the ones in the Tittabawassee River with respect to their profiles. Thus dioxin contamination may be playing a role in the increase in breast cancer incidence within these clusters, though other factors cannot be ruled out. This hypothesis underscores the need for more dioxin sampling efforts in these areas. The detection of ZIP codes 48457 (Figure 6) and 48734 (Figures 5 and 6) as spatial clusters may be in part due to their small at-risk background populations (4,164 and 3,924 females in 2000 respectively). Small population problem causes an area with a small population to be less reliable due to the higher variance. This is prevalent in rare disease analysis, especially in cancer studies when rates are used to estimate the underlying risk [52].

The findings in this study are subject to at least four limitations. First, the sparsity of soil dioxin data and scale of the breast cancer incidence data may have introduced uncertainties into health outcomes. The lack of TEQ data for other soils from background sites/ZIP codes and locations farther away from Midland were limiting factors, therefore we could not definitively confirm spatial clusters that are located farther away. The number and distribution of soil samples clearly were not sufficient to ascertain the contamination range, yet this dioxin database is the most comprehensive in the study area to date. Second, the ZIP code of residence at diagnosis is inadequate to describe an individual's location during the development of cancer. This surrogate for exposure is insufficient especially when causative exposures occur largely in areas other than residence locations, such as in areas related to occupational or recreational activities. Further analysis should include characterization of environmental exposure and cancer risk at the individual level. Third, the data sets lacked residential history information. Breast cancer is known to have long latencies [26,35,49]. The time when the patient was diagnosed may not be the time when causative exposures occurred. In addition, the migration during the latencies tends to obscure relationships between environmental exposure and cancer incidence [35]. Yet the information about residential history is restricted because of privacy concerns. Fourth, this study was not able to fully adjust all confounding risk factors of breast cancer development. We considered age effect; however, we did not adjust for other confounders, such as each patient's race, childbearing patterns, socioeconomic status, exposure to other pollutants because some of the information is not available to the public. Yet they are substantive factors in the development of breast cancer [22,38,53-55]. In a separate follow-up study [56], we have critically evaluated the spatial clusters established in this study and environmental pollutants.

Although the association between increased incidence of breast cancer and living on or close to dioxin contamination areas was found in our study, the question of whether exposure to dioxin in soil has caused or is causing breast cancer in this region is obviously complex and likely to be answered only through various comprehensive approaches and by controlling for other confounders. For example, in a separate report [56] we compiled more than 325 chemicals that are released into the environment besides dioxins. It is possible that these chemicals contribute to the high rates of breast cancer as well.

## Conclusion

In summary, this study finds that there are elevated levels of dioxin contamination in the city of Midland and Tittabawassee River 100-year floodplain. We identified a spatial association between greatly elevated levels of breast cancer incidence rates in city of Midland and contaminated areas. The spatial clusters of breast cancer incidence rates near contaminated areas suggest that there are important factors that contribute to the disease burden among women that must be fully investigated in future research. Although these findings are not sufficient to establish the causal relationship between exposure to dioxin and the development of breast cancer, they are important for formulating new hypotheses regarding the dioxin contamination and incidence of breast cancer in this study region.

## Competing interests

The authors declare that they have no competing interests.

## Authors' contributions

Both authors have made substantive contributions to this study. DD and TJO conceived this study. DD designed the study and developed the study's genetic algorithm models. He assembled and analyzed the data, performed the spatial and statistical analysis, and produced the first draft of this manuscript. TJO provided assistance on data acquisition and data interpretation, participated and adviced on the design of the study, methods, and analysis, as well as edited several drafts of the manuscript. Both authors have read and approved the final manuscript.
